# Design and application of self-healable polymeric films and coatings for smart food packaging

**DOI:** 10.1038/s41538-023-00185-3

**Published:** 2023-03-29

**Authors:** Wing-Fu Lai

**Affiliations:** Department of Applied Biology and Chemical Technology, Hong Kong Polytechnic University, Hong Kong Special Administrative Region, China

**Keywords:** Polymers, Polymer characterization

## Abstract

Smart packaging materials enable active control of parameters that potentially influence the quality of a packaged food product. One type of these that have attracted extensive interest is self-healable films and coatings, which show the elegant, autonomous crack repairing ability upon the presence of appropriate stimuli. They exhibit increased durability and effectively lengthen the usage lifespan of the package. Over the years, extensive efforts have been paid to the design and development of polymeric materials that show self-healing properties; however, till now most of the discussions focus on the design of self-healable hydrogels. Efforts devoted to delineating related advances in the context of polymeric films and coatings are scant, not to mention works reviewing the use of self-healable polymeric materials for smart food packaging. This article fills this gap by offering a review of not only the major strategies for fabrication of self-healable polymeric films and coatings but also the mechanisms of the self-healing process. It is hoped that this article cannot only provide a snapshot of the recent development of self-healable food packaging materials, but insights into the optimization and design of new polymeric films and coatings with self-healing properties can also be gained for future research.

## Introduction

Packaging films and coatings can be used to protect a food product from the harsh environment, and to provide ingredient and nutritional information about the food^1,2^. Till now different types of materials have been adopted for food packaging applications. Examples of these include metals (e.g., aluminium foil and tinplate), glass and polymers^3–8^. Among them, polymers are the most extensively studied group partly because of their high structural flexibility and property tunability^9–12^. For example, starch films have been reported as edible clusteroluminogenic films to package frozen foods^13^. Food-grade polyelectrolyte complex films generated via electrostatic interactions between hypromellose-graft-chitosan and carboxymethylcellulose sodium (CMC) have also been designed for food packaging applications^14^. The films exhibit good barrier properties and high transparency. Owing to the antibacterial properties of the chitosan (CS) moiety, the films can inhibit the growth of both Gram-negative and Gram-positive bacteria^14^. Importantly, the physical properties (including the mechanical strength and water vapour permeability) of the films can be tuned easily by manipulating the film constituents^14^. Such high tunability in the properties of a polymeric film has also been observed in a film generated from a cellulose derivative^15^. The UV-shielding capacity and wettability of the film can be altered simply by changing the molecular weight of the derivative or the concentration of the film-forming solution^15^. This enables the properties of the film to be tuned to meet different needs of the food product to be packaged.

Despite the advances in the development of packaging materials and in optimization of material properties as mentioned above, proper functioning of the packaging materials is secured only when those materials remain intact. The food package may, however, be damaged during storage and transportation, leading to an irreversible decline in the ability of the package to protect the food from outside influences^16–19^. To address this problem, one strategy is to render the film and coating self-healable. Over the years, extensive efforts have been devoted to the development of self-healable polymeric materials; however, most of the discussions made in the literature are confined to self-healable hydrogels^20–24^. Efforts devoted to examining related advances in the context of polymeric food packaging films and coatings are scant. In order to fill this gap, the objectives of this article are to review existing strategies for fabrication of self-healable polymeric packaging films and coatings, and to revisit major mechanisms of the self-healing process. It is hoped that by reviewing the recent development of self-healable food packaging materials, insights into the optimization and design of smart packaging materials with self-healing properties can be attained for future research.

## Mechanisms of the self-healing process exhibited by smart packaging materials

As far as self-healing of films and coatings is concerned, it can be extrinsic or intrinsic. Extrinsic self-healing is achieved mainly by adding a healing agent, which is embedded in capsules or vascular networks, into the polymer matrix during the film-forming process^25^. Inclusion of capsules or vascular networks, however, may reduce the mechanical strength of the generated film and coating. Along with the instability of the healing agent, applications of extrinsic self-healing are limited. An alternative to extrinsic self-healing is the intrinsic one, which relies on reversible interactions within the polymer matrix to achieve the self-healing process upon the presence of an external stimulus. It is mediated mainly by chain movement and recombination^26^, without being needed to be driven by any external healing agent prefilled in capsules or vascular networks. The possible integration of intrinsic self-healing into the design of a packaging material is partially demonstrated by the case of hydrophobic films generated from cellulose and natural wax^27^. The films can be obtained by first dispersing natural wax emulsion latex in an iced alkali/urea aqueous system, in which cotton linter pulp is also dissolved, to generate a cellulose/natural wax suspension^27^. Upon the process of solution casting and annealing treatment, the suspension is converted into films showing high tensile strength, good hydrophobicity, and good biodegradability^27^. After annealing treatment at elevated temperature to stimulate the release of wax from the inside to the surface of the cellulose matrix, damage in the films can be healed^27^. In addition, upon treatment with humidity, poly(vinyl alcohol) (PVA)-based packaging films containing layered double hydroxide (LDH) nanoplatelets and poly(sodium styrene-4-sulfonate) (PSS) can undergo self-healing upon damage^28^.

Intrinsic self-healing can be made possible by incorporating dynamic molecular interactions into the matrix of the packaging material (Table 1)^29–37^. One type of these interactions is Schiff base formation, which has been reported to be achieved when CS interacts with dialdehyde-modified cellulose nanocrystals (CNCs)^38^, zinc phthalocyanine tetra-aldehyde^39^, vanillin^40^ and dialdehyde starch^41^. Other examples of reversible interactions include hydrogen bonding interactions and electrostatic interactions. They can be obtained when CS interacts with poly(acryloyl-phenylalanine)^42^, CMC^43^, sodium alginate (SA)^44^, and poly(acrylic acid)^45^. So far electrostatic interactions are the most extensively adopted interactions exploited for the design of self-healable food packaging films. To achieve such interactions, a polycation and a polyanion are often involved during film fabrication. One good example is the polyelectrolyte multilayered edible film generated from negatively charged CMC and positively charged CS via layer-by-layer (LbL) electrostatic deposition^43^. Due to the antibacterial properties of CS, the film can inhibit the growth of Gram-negative bacteria^43^. The scratched film can undergo self-repair upon the addition of water to the damaged region^43^. By using freshly-cut apples as a model, the film has demonstrated good capacity in inhibiting the enzymatic browning process and the weight loss of the apples^43^. All these reveal the application potential of the film in fruit preservation.

**Table 1:** Major types of interactions used in the design of self-healable materials.

Type of interactions	Nature of interactions	Mechanism	Example of application	Ref.
Covalent interactions	Boronate ester bonds	Bonds formed by complexation of boronic acid and diols	A gel has been generated by mixing phenylboronic acid with diol-modified polyethylene glycol. The self-healing ability of the gel is attributed to the dynamic formation of boronic esters between phenylboronic acid and *cis*-diols.	^29^
	Disulphide bonds	Bonds formed by two sulphur-containing atoms	A gel has been generated based on the thiol/disulphide exchange of thiol-functionalized F127 and dithiolane-modified polyethylene glycol. The self-healing ability of the gel is attributed to the reversible exchange of the free thiols and the reformation of dithiolane rings.	^30^
	Imine bonds	Bonds formed by the reaction between a carbonyl compound and an amino group, with water being eliminated during the process	A gel has been formed by using N-carboxyethyl CS (CEC) and dibenzaldehyde-terminated polyethylene glycol (PEGDA). The self-healing ability of the gel is attributed to the presence of dynamic covalent Schiff-base linkages between amine groups of CEC and benzaldehyde groups of PEGDA.	^31^
	Coordination bonds	Bonds formed by sharing an electron pair from a single atom	Metal-ligand coordination bonds between cerium ions and conjugated 8-hydroxyquinoline have enabled a polymeric film to undergo the process of self-healing.	^32^
	Diels-Alder reactions	Reactions between conjugated dienes and alkenes to form unsaturated rings	A dually cross-linked SA-based gel has exhibited self-healing capacity due to the dynamic nature of the Diels-Alder cross-links formed by a four-arm polyethylene glycol cross-linker as well as the reversibility of the ionic cross-links formed by calcium ions.	^33^
Non-covalent interactions	Hydrogen bonds	Reversible interactions between hydrogen atoms and electronegative atoms	Watson-Crick base pairing between the nucleobases through hydrogen bonds has been exploited in the generation of a self-healable gel formed by using cytosine- and guanosine-modified hyaluronic acid.	^34^
	Host-guest interactions	Complexation of two molecules or agents through specific structural relationships and noncovalent interactions	A bionic self-healable waterborne polyurethane elastic film has been generated based on cyclodextrin–ferrocene host-guest interactions. The re-establishment of reversible host-guest interactions upon film damage partly contributes to the healing of the film.	^35^
	Hydrophobic interactions	Interactions led by aggregative hydrophobes in an aqueous medium	A gel has been generated based on surfactant-free hydrophobic interactions, which can be obtained by evaporating the solvent from an aqueous polymer solution so as to increase the polymer concentration above the critical level.	^36^
	Electrostatic interactions	Interactions formed between oppositely charged molecules	A film has been generated via random copolymerization of [2-(methacryloyloxy)ethyl]dimethyl-(3-sulfopropyl)ammonium hydroxide (MSA) and 2-acrylamide-2-methylpropanesulfonic acid. The self-healing capacity of the film is partially attributed to electrostatic interactions among the MSA units.	^37^

Another type of interactions used in intrinsic self-healing is hydrogen bonding. The possible use of this in the design of self-healable packaging materials has been shown by the case of a zwitterionic anti-fogging coating generated from (methacryloyloxy)ethyldimethyl-(3-sulfopropyl), which is copolymerized by itaconic acid via the process of radical polymerization^46^. Because of the presence of hydrophilic groups in (methacryloyloxy)ethyldimethyl-(3-sulfopropyl), the coating shows anti-fogging capacity^46^. In addition, itaconic acid plays a role in mediating formation of reversible non-covalent hydrogen bonding interactions in the coating, rendering the coating self-healable^46^. Importantly, the (methacryloyloxy)ethyldimethyl-(3-sulfopropyl) moiety can adsorb water molecules, producing a protective layer on the coating surface to prevent adsorption of hydrophobic contaminants^46^. This allows the coating to show good self-cleaning properties. Besides Schiff base formation and hydrogen bonding interactions, host-guest interactions have been exploited during the design of self-healable food packaging materials. This is demonstrated by the case of the coating generated from SA and l-menthol-β-cyclodextrin-graft-CS^47^. During coating fabrication, β-cyclodextrin (β-CD) is first diluted in dimethyl sulfoxide and isobutyl alcohol, followed by the addition of sodium hydroxide and epichlorohydrin. Upon the addition of CS, β-CD-grafted CS is formed. It is then dissolved in water, and mixed with an alcoholic solution of l-menthol to form a complex solution. The solution is spin-coated on a glass substrate pre-coated with an aqueous solution of SA. The multi-layered coating is formed after multiple cycles of deposition. Incorporation of l-menthol has been found to increase intermolecular interactions among molecules inside the coating, leading to formation of a denser coating structure and hence an increase in the water vapour barrier capacity^47^. In addition, the incorporation of l-methanol makes the coating more hydrophobic and transparent^47^. The self-healing efficiency of the coating has been found to increase from 52.56% to 59.49% after the addition of l-menthol^47^. This is largely because the presence of l-methanol enables the damaged coating to undergo not only electrostatic interactions between polymer chains but also host-guest interactions between β-CD and l-menthol. This enhances the self-healing capacity of the coating.

## Strategies for fabrication of self-healable food packaging materials

**Fig. 1:**
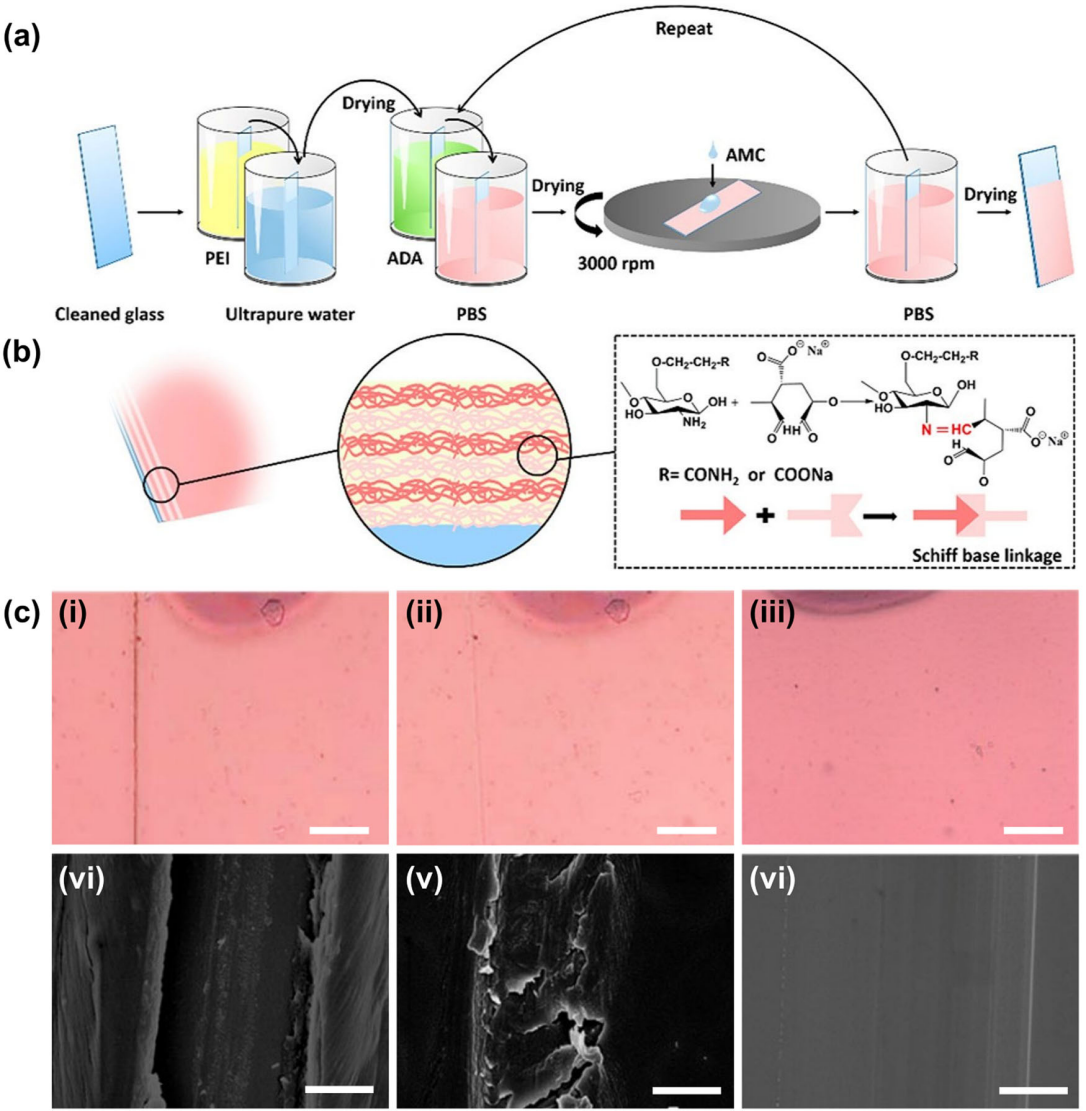
Fabrication and self-repair of the ADA/AMC film. **a** A schematic diagram illustrating the process of ADA/AMC film preparation; **b** Mechanism of formation of the Schiff base linkage; **c** (i–iii) Microscopic and (iv–vi) SEM images of the ADA/AMC film exhibiting self-healing: (i, iv) fresh cut, (ii, v) self-healing for 1 min, and (iii, vi) self-healing for 2 min. The film has undergone six cycles of deposition. The scale bar in (i), (ii) and (iii) represents 100 µm. The scale bar in (iv), (v) and (vi) represents 5 µm. Reproduced from 48 with permission from American Chemical Society.

Over the years, different strategies (ranging from solvent casting and extrusion to dipping and panning) have been reported for generation of polymeric films and coatings (Table 2)^32,48–53^. As far as self-healable films and coatings are concerned, the LbL assembly approach is, however, the most commonly adopted one. This may be partially because the LbL assembly procedure is easy to be performed and allows the properties (e.g., the thickness of the film and the composition of each layer) to be precisely controlled, thereby enabling the performance of the self-healable film and coating to be better fine-tuned. By using this method, transparent films have previously been generated from acrylamide-modified CS (AMC) (having a degree of substitution of 0.54) and alginate aldehyde (ADA) (having an oxidation degree of 50%) via formation of the Schiff base linkage^54^. During film fabrication, polyethylenimine (PEI) is first pre-deposited on a glass slide (Fig. 1), which is pre-treated with a Piranha solution to endow it with negative surface charge. This process can facilitate the subsequent LbL assembly procedure^54^. The slide pre-deposited with PEI is immersed in an aqueous solution of ADA, followed by being rinsed in phosphate-buffered saline (PBS)^54^. An aqueous solution of AMC is coated on the slide via the process of spin-coating^54^. After the removal of unadsorbed AMC molecules, the first deposition cycle of the film is complete^54^. Characterization by ultraviolet-visible spectroscopy and field emission scanning electron microscopy reveals that the film undergoes linear growth during the LbL process^54^. The thickness of the film is in the range of 0.6 ± 0.10 μm (3 bilayers) to 2.7 ± 0.05 μm (15 bilayers)^54^. Once the film is damaged by using a razor blade and drops of water are added to the damaged zone, the crack fades upon 1 min and disappears after 2 min. This self-healing process is hypothesized to be caused by water-induced swelling of the film and the high mobility of the polymer network^54^. Both of these factors are expected to play a role in facilitating the reconstruction of the Schiff base linkage to heal the damaged film.

**Table 2:** Major approaches of film and coating fabrication.

Type	Method	Working mechanism	Example of application	Ref.
Film formation	Extrusion	A film is formed from a polymer through either a blown process or a cast process	A high-amylose corn starch edible film has been produced via the extrusion process for packaging mangoes.	^48^
	Solvent casting	A film is formed by evaporating the solvent from a film-forming solution, which is put on a flat substrate	A composite film consisting of CS and banana peel extract has been generated by using solvent casting to improve the postharvest quality of apple fruits.	^49^
	LbL assembly	A multi-layered film is formed by depositing multilayers of polymers on a solid substrate	A CS/SA-based multi-layered film containing cinnamon essential oil has been generated by using the LbL assembly approach for controlling penicillium expansion of wounded apples.	^50^
	Spin coating	A film is formed by spreading a film-forming solution on a flat substrate by using the centrifugal force	A self-healable film consisting of responsive polymers has been generated by spin-coating the polymer solution on an aluminium substrate at 150 rpm for 300 s with a ramping acceleration of 70 rpm·s^−1^.	^32^
Coating formation	Dipping	A coating is formed by dipping a substrate into a coating solution	A SA-based edible coating has been applied to plum fruits via the process of dipping so as to preserve fruit quality during postharvest storage.	^51^
	Spraying	A coating is formed by spraying a coating solution on the surface of a substrate	A bovine gelatin solution has been spray-coated onto beef tenderloins, pork loins, salmon fillets, and chicken breasts for food preservation.	^52^
	Panning	A coating is formed on the surface of a food product by using a spinning bowl, in which a layering solution is drizzled or sprinkled.	A chocolate coating has been applied to rice crisp balls via the process of panning. The same process has been adopted to coat the chocolate panned product with an edible coating consisting of hydrolysed collagen, sucrose and cocoa butter.	^53^

Since the turn of the last century, deep eutectic solvents have been employed for fabrication of self-healable films. These solvents were first reported in early 2000’s when a mixture of urea and choline chloride was found to be in the liquid state at ambient conditions^55^. Owing to charge delocalization caused by hydrogen bonding interactions between urea molecules and chloride ions, the mixture melts at a temperature lower than the melting point of each of the constituents^55^. Deep eutectic solvents show various favourable properties such as high chemical stability, non-flammability, high thermal stability and low volatility^55^. In addition, by modulating the molar ratio of different constituents, the properties of the deep eutectic solvent can be tuned^55^. Over the years, deep eutectic solvents have been used as modifiers of diverse polysaccharides (ranging from starch^56^ to guar gum^57^) owing to their capacity of forming a network of hydrogen bonds. Recently, a deep eutectic solvent, which consists of citric acid and choline chloride, has been used to combine with CS to generate a self-healable film^58^. Owing to the dynamic network of hydrogen bonds between CS and the components of the deep eutectic solvent, the mechanical strength of the generated film can be recovered after damage^58^. This demonstrates the possible role played by deep eutectic solvents in the design of self-healable films and coatings for food packaging.

## Optimization and engineering of self-healable food packaging materials

**Fig. 2:**
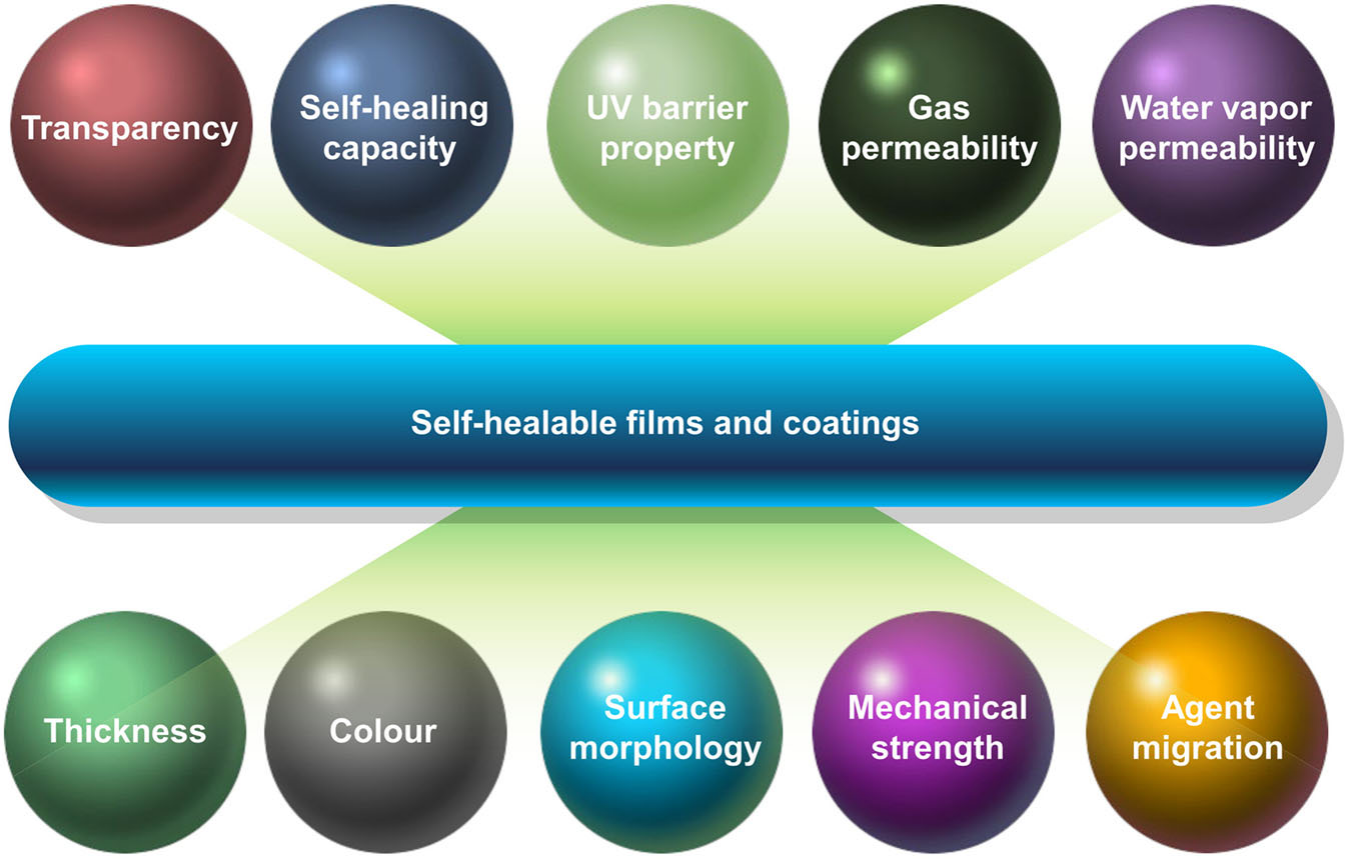
Some major factors to be determined in evaluation of self-healable packaging films and coatings.

In order to achieve the optimal performance of a self-healable film and coating, proper consideration of multiple factors is required (Fig. 2). One factor to be considered during the design of self-healable food packaging materials is the thickness of the film and coating. It may affect the number of times the damaged packaging material can be self-healed. This is demonstrated by the case of AMC/ADA films^54^. While films with different thickness show no significance difference in their healing time, the number of healing cycles increases as the thickness of the films increases^54^. This is due to the fact that a thicker film contains more free amino groups and aldehyde groups for reconstruction of the Schiff base linkage to heal the damaged area^54^. Another important factor to be considered is the chemical nature of the film constituent. Compared with coatings generated from proteins and lipids, those generated from polysaccharides are comparatively stable in performance and can be produced in a lower cost due to the natural abundance of polysaccharides^59^. In addition, incorporation of a nanobrick wall structure, in which layered materials and polymers are stacked with nanoplatelets, into a film or coating has been shown to enhance the performance of the generated product^60^. Common examples of nanoplatelets used for this purpose include graphene, LDH, and montmorillonite (MMT)^60^. Among them, MMT has attracted great interest because of its low cost and its high dispersibility in polymer solutions. Recently, Manabe and co-workers have developed a cephalopods-inspired self-healable nanoclay composite coating for food packaging applications^60^. The coating is an MMT-containing ion-doped multilayer membrane composed of branched PEI (bPEI) and poly(acrylic acid) (PAA). Not only can the presence of the counterions reduce the charge density of the polymer but it can also convert the conformation of the polymer from a loop structure to a train structure^60^. This helps improve the capacity of the coating in water absorption and retention^60^. In addition, the LbL approach allows the thickness of the coating to be finely tuned^60^. The process of self-healing is initiated upon the immersion of the coating into water^60^. Compared with the conventional non-ion-doped polymer multilayer in which the carboxyl groups of PAA dissociate completely and form bonds with PEI, the MMT-containing ion-doped counterpart enables repair of damage to be completed within a much shorter timeframe^60^. This is attributed to the fact that the addition of counterions and MMT inhibits formation of bonding interactions among polymer chains in the multilayer, thereby enhancing the mobility of the polymer chains during the self-healing process.

When a food packaging film is designed, bonding interactions involved in film formation and repair play a principal role in determining the ultimate properties of the film generated. In general, non-covalent interactions are weaker than covalent bonds^61,62^. Using different dynamic non-covalent bonding interactions to replace the conventional covalent ones in film design may result in a decline in the mechanical strength of the generated film. The trade-off between the self-healing capability and the mechanical strength, therefore, has to be considered. Among diverse film properties, the barrier properties, particularly gas permeability, are crucial to be taken into account during film design. In fact, oxidation in food products is one of the biggest problems to be addressed during food preservation^63–68^. This problem is particularly serious in food products (such as fish oils, nuts, and fishery products) rich in fats^69^. Lipid oxidation is one of the major processes leading to deterioration of the sensory attributes of a food product^70,71^. It leads to formation of toxic aldehydes, and causes a loss of nutritional values^72^. This problem is particularly severe in food products that possess a high content of polyunsaturated fatty acids (PUFAs)^1^, which are susceptible to oxidation reactions that can be initiated once a hydrogen atom is abstracted from an unsaturated fatty acid to produce an alkyl radical^73^. Because the initiation reaction is not thermodynamically favourable, an alkyl radical is generated mainly in the presence of pigments (which serve as photoensitizers) or singlet state oxygen^74^. The latter is produced when a food product is exposed to UV light or temperature change^74^. In addition, the generated alkyl radical can react with O_2_ to produce a peroxyl radical, which can abstract a hydrogen atom from an unsaturated fatty acid to generate a new alkyl radical and a lipid hydroperoxide^75^. The lipid hydroperoxide is tasteless and odourless; however, in the presence of light and heat, it can decompose to compounds that produce off-flavours. A large variety of volatile compounds (including alkenes, alkanes, aldehydes, and alkanals) are also generated during the process of lipid peroxidation. Some of these compounds (such as dimethyl disulphide, pentanal, hexanal, 3-hydroxy-2-butanone, 2-hexenal, butanoic acid, methanethiol, nonenone, 2-nonenal, and dimethyl trisulfide) can give off-odours^73,74^. To retard oxidative deterioration of a food product, one strategy is to lower the localized concentration of oxygen in a food package. Over the years, a variety of packaging materials showing low oxygen permeability have been designed^76–78^. Despite this, long-term durability is a problem that has yet to be fully solved. Influx of oxygen will occur when the films and coatings are flexed or when a crack is formed. This reduces the shelf life of the food product.

**Fig. 3:**
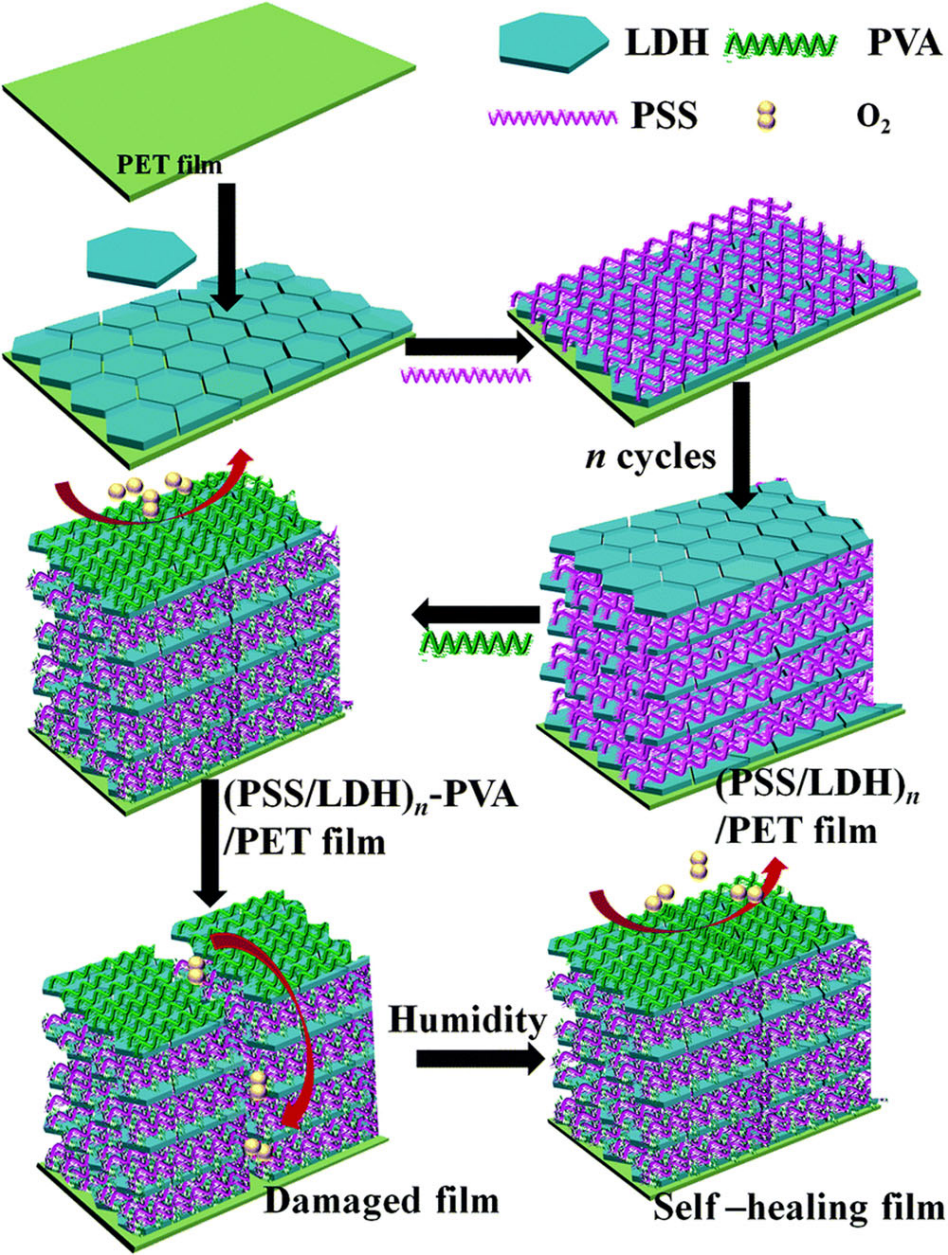
The process of fabrication of a humidity-triggered self-healable film on a polyester substrate. Reproduced from 28 with permission from Royal Society of Chemistry.

The possibility of manipulating the barrier properties of a self-healable food packaging material has been revealed by a recent study, in which humidity-triggered self-healable films with excellent oxygen barrier performance have been developed using the LbL self-assembly technique (Fig. 3)^28^. During film fabrication, a substrate is first dipped into a colloidal suspension of LDH nanoplatelets, followed by a washing step before the substrate is further immersed in an aqueous solution of PSS. After alternate deposition of LDH nanoplatelets and PSS for a number of cycles, the LbL films are produced. The films are then immersed in a PVA solution to enable PVA molecules to permeate into the films. Proper optimization of the process of PVA permeation is vital to enable the generated films to show high self-healing capacity^28^. While the self-healing performance will be poor if the PVA coating is too thin, the healing time needed by the films will be very long if the coating is too thick^28^. In addition, PVA permeation causes the films to have a more compact structure, leading to a decrease in the oxygen transmission rate (OTR)^28^. Upon flexing, the OTR of the films increases. Yet, the oxygen diffusion resistance of the films can be recovered after being put in an environment with the humidity of around 85%^28^. This demonstrates the humidity-triggered self-healing capability of the films.

## Applications of self-healable polymeric materials in smart food packaging

**Fig. 4:**
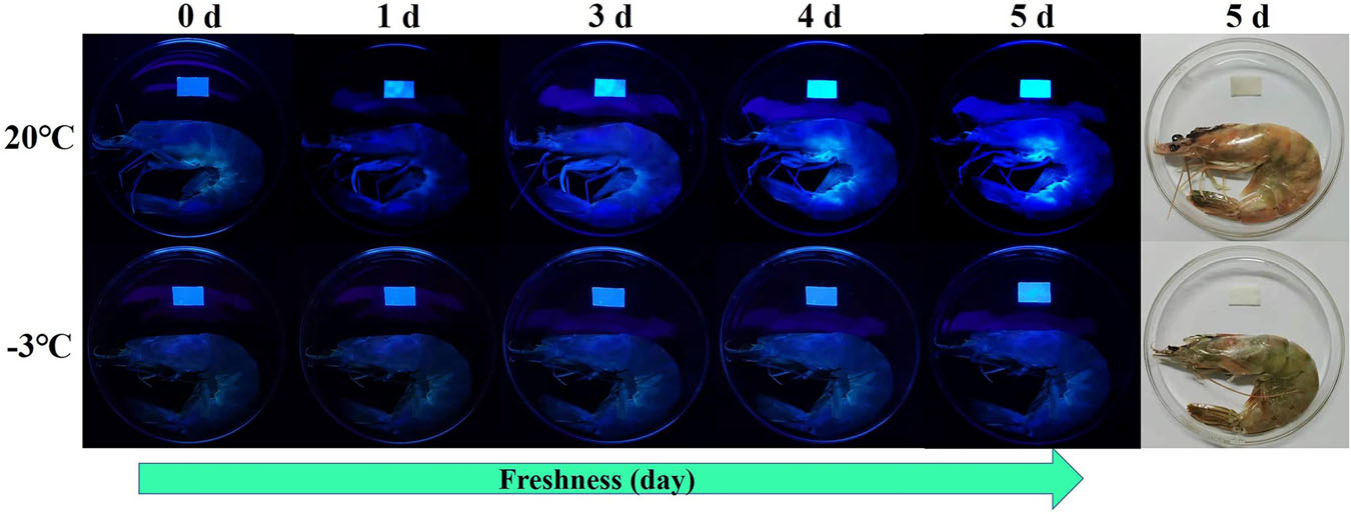
Fluorescence images of the self-healable polyurethane films applicable to shrimp packaging. The fluorescence of the films enables real-time monitoring of the freshness of shrimps stored at different temperatures. Reproduced from 79 with permission from Elsevier B.V.

Films and coatings can be applied to package different food types, ranging from seafood to fruits and vegetables. For example, the self-healable polyurethane films, in which diacetyl oxime and coumarin diol serve as the reactive filler, have been applied to package shrimps, with their fluorescence intensity and colour being able to function as a sensor for real-time and visual monitoring of the freshness of seafood (Fig. 4)^79^. Edible self-healable coatings generated from SA and l- menthol-β-cyclodextrin-graft-CS have also been used to improve the post-harvest quality and to prolong the shelf life of fruits and vegetables^47^. During fruit preservation, the fruit is first immersed in a hypochlorite solution for 30 s before being flushed with distilled water. It is then immersed in a SA solution for 6 min, followed by immersion in a clathrate complex solution for 6 min. After the coating is air-dried at ambient conditions, another cycle of the coating process is conducted. In total 10 deposition cycles are performed. After 30 days of storage, the uncoated fruit has been reported to have a weight loss of around 15% whereas the coated one has only had a weight loss of 7.6%^47^. Compared to the uncoated counterpart, the coated one has also been found to be 15.23 % higher in its firmness^47^. All these illustrate the effectiveness of self-healable packaging materials in preserving different types of food products.

**Fig. 5:**
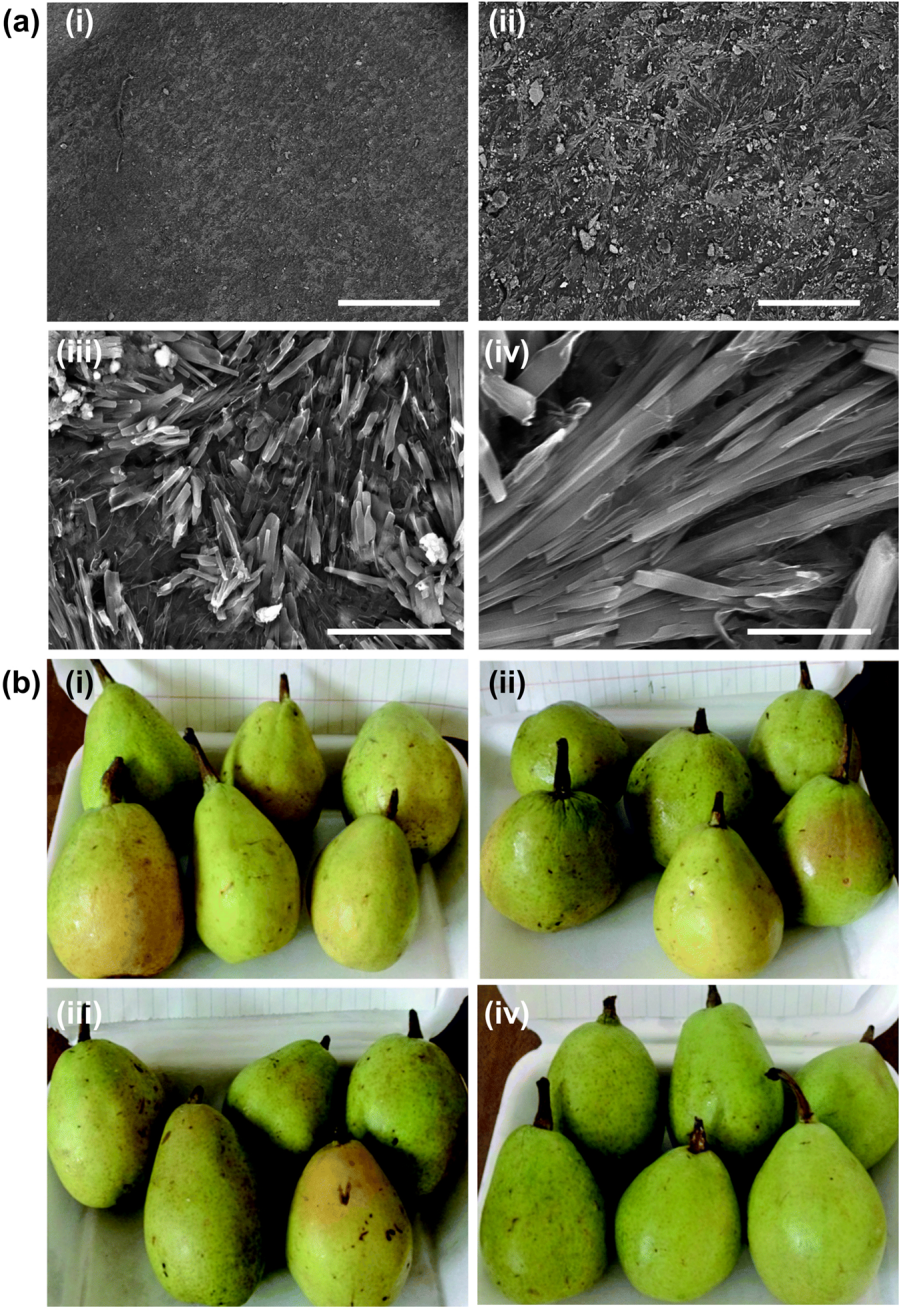
Surface morphology and performance of the CS-beeswax/pollen grains coating in fruit preservation. **a** Scanning electron microscopic images of the CS-beeswax/pollen grains coating at various magnification scales. The scale bar in (i), (ii), (iii) and (iv) represents 1 mm, 200 µm, 30 µm and 10 µm, respectively. **b** Appearance of (i) uncoated pears, (ii) pears coated with CS alone, (iii) pears coated with a mixture of beeswax and pollen grains, and (iv) pears coated with both CS and beeswax-pollen grains. The pears have been stored at 0 ± 1 °C for 105 days and at 23 ± 2 °C for 1 week. Reproduced from 80 with permission from Royal Society of Chemistry.

Despite the practical potential of self-healable packaging materials as mentioned above, till now self-healable films and coatings reported in the literature have been examined mostly in fruit and vegetable packaging. This may be partially because fruits and vegetables are fragile products with high water content. Along with the fact that changes in their quality can be easily detected based on their appearance and weight change, fruits and vegetables serve as favourite models in evaluating the performance of a food packaging film. Over the last several decades, different films possessing self-healing capacity have been exploited to package fruits and vegetables. For instance, by using a 3-layered film consisting of CS and CMC to coat lemon fruits (*Citrus aurantifolia* Swingle), the pectinase enzyme activity (as well as the loss of vitamin C) of the coated fruits has been shown to be much lower than that of the uncoated ones^80^. The application potential of self-healable polymeric films and coatings in postharvest preservation of fruits has been further demonstrated by the case of the smart edible coating generated from CS and beeswax-pollen grains^81^. During coating preparation, beeswax, glycerol, pollen grains and tween 80 are added to an acetic acid solution containing CS, glycerol and calcium chloride dihydrate^81^. The presence of edible hydrophobic components such as beeswax and pollen grains help improve the physical and barrier properties of the CS-based coating^81^. Once the coating is damaged, calcium ions can diffuse along the interface of the cut points and help re-establish dynamic supramolecular interactions (including electrostatic interactions and hydrogen bonding interactions) to heal the damaged area^81^. The self-healing efficiency of the coating is estimated to be around 80–90%^81^. By using Le Conte pears as a model, the weight loss percentage of the coated fruits under cold storage conditions has been found to be much less than that of the uncoated counterparts^81^. Owing to the ability of the coating to restrict gas exchange through the peel and to inhibit ethylene gas generation, the coated pears are more resistant to decay as compared to the uncoated ones^81^. The coating can also inhibit water loss and the activity of pectin-degrading enzymes^81^. This retards the rate of fruit softening. Compared to the uncoated pears or those coated with either CS or a mixture of beeswax and pollen grains, those coated with both CS and beeswax-pollen grains exhibit better appearance and an increase in the total soluble solid percentage (Fig. 5)^81^.

Recently, one multi-layered coating showing self-healing capacity has been developed to extend the shelf life of fruits^44^. During coating formation, a substrate is repeatedly immersed in an acetic acid solution of CS and an aqueous solution of SA^44^. Molecules of CS and SA in the coating are linked to each other via both hydrogen bonds and ionic bonds^44^. Microscopic examination reveals that the coating has a smooth surface, with the hydrophobicity of the coating depending largely on the properties of the outer layer but not on the interactions between SA and CS^44^. In addition, the degree of mobility of the polyelectrolyte multilayer is influenced by the number of deposition cycles during coating formation^44^. Healing of the coating can be initiated upon the addition of water, which can accelerate the movement of molecules between layers of the coating. This enables the mechanical strength, oxygen barrier capacity and water vapour barrier properties of a damaged coating to be recovered^44^. The performance of the coating in fruit preservation has been evaluated by using strawberries as a model. Five days after the coating process, spoilage has been found to be lower in coated strawberries than in the uncoated ones. This is partly due to the antibacterial activity of CS. The coating has also been shown to inhibit water loss from the strawberries, and to lower the content of malondialdehyde (MDA), which is a secondary end-product of membrane lipid peroxidation and can serve as an indicator of oxidative stress^82^. Importantly, while damage of the coating can lead to an increase in the water vapour permeability and oxygen permeability and hence an accumulation of MDA, such effects have been successfully reduced upon the healing of the coating.

## Opportunities and challenges for practical applications

When films and coatings are generated, various additives may be added to manipulate the properties and to increase the functionality. For example, CNCs have been extracted from cotton textile waste and modified with methacrylamide (MAM)^83^. Composite films, consisting of cellulose acetate and poly(vinyl chloride), have been found to show higher mechanical and UV barrier properties upon the incorporation of MAM-CNCs^83^. After chlorination, MAM-CNCs also enable the composite films to act against both *Staphylococcus aureus* ATCC 6538 and *Escherichia coli* K12^83^. Recently, incorporation of quaternized CNCs into PVA films as nanofillers has been reported to give the films higher tensile strength and higher antibacterial activity^84^. Apart from nanofillers as mentioned above, plasticizers can be added to help weaken intermolecular interactions among polymer chains^85^, thereby reducing the tension of deformation exhibited by the film, improving the polymer chain flexibility^86–90^ and increasing the resistance of the generated film to fracture^85^. Examples of commonly used plasticisers include polyols (e.g., sorbitol, polyethylene glycol, and glycerol) and lipids^85^. Despite the possible use of these agents in improving film properties, migration of these chemicals from a packaging film to a food product may lead to not only concerns on toxicity but also changes in the sensory attributes and quality of the food. Migration is defined as the mass transfer of substances from the packaging material to the packaged food^91^. It is one of the major safety concerns in food packaging. As stated by the European Union (EU) regulations, there should be no constituent of a food contact material that can transfer to the packaged food in an amount that can either endanger the health of the consumer or deteriorate the organoleptic features of the food^91^. In order to determine the safety profile of a packaging film and coating, migration tests can be conducted. However, such tests are time-consuming and laborious^91^. The accuracy of such tests is also hampered when proper analytical methods to determine the migrant are lacking^92^. This problem is compounded by the fact that compounds added to the film and coating may react with other packaging material constituents or food components, leading to formation of new chemical entities that are overlooked when detecting potential migrants. In addition, different food compositions and storage conditions may cause changes in the level of migration of substances from the packaging material to the packaged food^91^. All these make eradication of safety concerns on the generated films and coatings difficult, thereby impeding the practical use of those films and coatings in practice. To enhance the accuracy of migration tests, apart from using the actual conditions of storage of the real food product as the experimental conditions used in migration tests, the food model adopted should be the same as the food product to be packaged by using the film and coating under investigation. More sophisticated methods have to be developed to predict products formed via possible reactions between packaging material constituents and food components. Efforts are also needed to be paid to the development of analytical techniques with high sensitivity and specificity.

Apart from examining the safety of plasticizers, it is important to determine the number of tolerable damage-repair cycles that can be undertaken by a self-healable packaging material before the material is applied to package real products. However, most of the studies in the literature reporting the development of a self-healable polymeric food packaging material have only examined the efficiency of single-time healing. The capacity in multi-time healing has rarely been determined. This may be because, in the current approach to evaluate the self-healing performance, a cut on the material is often created manually by using scalpels or blades. The width and depth of the cut can hardly be precisely controlled, not to mention the difficulty of making the same cut in exactly the same position multiple times. This impedes the generated self-healable material to be translated from a laboratory to routine food packaging applications. Light to solve this problem has been shed recently by the emergence of colloidal lithography, which makes the generation of repeatable cuts with precisely controllable depth, width, shape, and location possible^93^. This approach has been reported to successfully generate cuts in the form of nanorods, nanowells, and microstripes on a self-healable PEI/PAA film^93^, enabling the limitation of a self-healable film and coating undergoing repeated damage to be studied.

Last but not least, right now most of the self-healable films and coatings reported in the literature have been evaluated only by using fruits and vegetables as the models. Here it is worth noting that films and coatings generated by using components (e.g., CS and CMC) of many of those self-healable counterparts have in fact been shown to be applicable to package many other food products. For example, CS films incorporated with *Cinnamodendron dinisii* Schwanke essential oil-loaded zein have been reported to successfully stabilize the deterioration reactions in ground beef^94^. Farsi gum-CMC films containing *Ziziphora clinopodioides* essential oil and lignocellulose nanofibers have also been exploited as packaging materials to extend the shelf life of fresh minced beef meat^95^. Although these packaging materials are not self-healable films and coatings, their success in packaging food products other than vegetables and fruits implicates the worth of exploiting the packaging performance of self-healable materials beyond mere fruit and vegetable preservation.

## Concluding remarks and future outlook

Self-healable packaging materials can maintain their protection of the packaged food even upon damage, and hence show practical values in both food product development and food preservation. In sections above, the latest status of the design and applications of self-healable films and coatings for food packaging have been presented. While several issues (including the development of more effective strategies to evaluate film migration and to determine the number of tolerable damage-repair cycles) have to be addressed to enhance practical translation of the generated films and coatings, some unmet needs in current research on self-healable packaging materials are also worth noting for future research. For example, at the moment the self-healing capacity of packaging materials have been evaluated in the literature predominately by using only simple qualitative approaches, in which the healing time and healing efficiency are determined simply under a microscope. Along with the fact that the depth, width and position of a cut made on the films and coatings in different studies are different, a fair comparison of the self-healing capacity of different reported packaging materials is basically impossible.

For future research, development of a more standardized and quantitative procedure to examine the self-healing performance is desired. This allows films and coatings generated in different laboratories to be fairly compared, thereby enabling more effective candidates to be selected for further development. In addition, at the moment self-healing of the reported food packaging materials largely requires the use of an external stimulus (ranging from humidity to elevated temperature) as a trigger. This implies that, if those films and coatings are used in a real situation, the self-healing process will function well only when any damage imposed on the films and coatings can be identified in real time and be immediately treated. This is obviously impractical. Development of films and coatings that can undergo automatic self-healing is, therefore, a direction that is worth exploration in future research. Clearly there is still some way to go before self-healable films and coatings can be routinely adopted in food packaging, but with the increasing sophistication of techniques in chemical synthesis and film optimization^96–101^, along with the merit brought about by the self-healing property, it is anticipated that self-healable materials will continue to contribute to food preservation in the forthcoming decades.

## Data Availability

Data sharing is not applicable. This is a review article and no new datasets were generated or analyzed during this article.
